# Targeting the JAK/STAT pathway in solid tumors

**Published:** 2020-08-21

**Authors:** Zoya Qureshy, Daniel E. Johnson, Jennifer R. Grandis

**Affiliations:** Department of Otolaryngology-Head and Neck Surgery, University of California San Francisco, San Francisco 94158, USA.

**Keywords:** Clinical trials, Janus kinase/signal transducer and activator of transcription (JAK/STAT) pathway, JAK inhibitors, solid tumors, STAT hyperactivation

## Abstract

Aberrant activation of signal transducer and activator of transcription (STAT) proteins is associated with the development and progression of solid tumors. However, as transcription factors, these proteins are difficult to target directly. In this review, we summarize the role of targeting Janus kinases (JAKs), upstream activators of STATs, as a strategy for decreasing STAT activation in solid tumors. Preclinical studies in solid tumor cell line models show that JAK inhibitors decrease STAT activation, cell proliferation, and cell survival; in *in vivo* models, they also inhibit tumor growth. JAK inhibitors, particularly the JAK1/2 inhibitor ruxolitinib, sensitize cell lines and murine models to chemotherapy, immunotherapy, and oncolytic viral therapy. Ten JAK inhibitors have been or are actively being tested in clinical trials as monotherapy or in combination with other agents in patients with solid tumors; two of these inhibitors are already Food and Drug Administration (FDA) approved for the treatment of myeloproliferative disorders and rheumatoid arthritis, making them attractive agents for use in patients with solid tumors as they are known to be well-tolerated. Four JAK inhibitors (two of which are FDA approved for other indications) have exhibited promising anti-cancer effects in preclinical studies; however, clinical studies specifically assessing their activity against the JAK/STAT pathway in solid tumors have not yet been conducted. In summary, JAK inhibition is a viable option for targeting the JAK/STAT pathway in solid tumors and merits further testing in clinical trials.

## INTRODUCTION

The Janus kinase/signal transducer and activator of transcription (JAK/STAT) signaling pathway is implicated in the development and progression of many cancers^[1,2]^. Hyperactivation of STAT transcription factors, has been reported in both hematologic malignancies and solid tumors, including cancers of the breast, lung, liver, head and neck, and stomach, among others^[3-8]^. For many of these cancers, increased activation of the JAK/STAT signaling pathway is associated with a worse prognosis, including increased recurrence and reduced overall survival^[1,9,10]^. Given the strong association between JAK/STAT hyperactivity and the development and prognosis of multiple cancers, STATs and their upstream activators, JAKs, are being extensively explored as targets for cancer therapy^[1,11-13]^.

Certain hematologic malignancies such as myeloproliferative neoplasms are associated with specific JAK mutations that serve as predictive biomarkers for JAK-targeted therapy^[14]^. The majority of cases of polycythemia vera, essential thrombocytopenia, and myelofibrosis are characterized by an activating valine to phenylalanine mutation in JAK2 (JAK2 V617F) that drives the development of these neoplasms^[15]^. Clinical trials studying the impact of ruxolitinib, a selective JAK1/2 inhibitor, on polycythemia vera and myelofibrosis demonstrated significant improvement in patient outcomes, leading to Food and Drug Administration (FDA) approval and widespread use of this agent for these diseases^[16-22]^. However, mutations in the JAK/STAT pathway are rare in solid tumors, and the role of JAK and/or STAT inhibitors for the treatment of solid tumors is incompletely understood. In this review, we describe the rationale for targeting the JAK/STAT pathway in solid tumors and summarize preclinical studies and clinical trials to date that evaluate the impact of agents targeting this pathway.

## JAK/STAT SIGNALING

**Figure 1.**
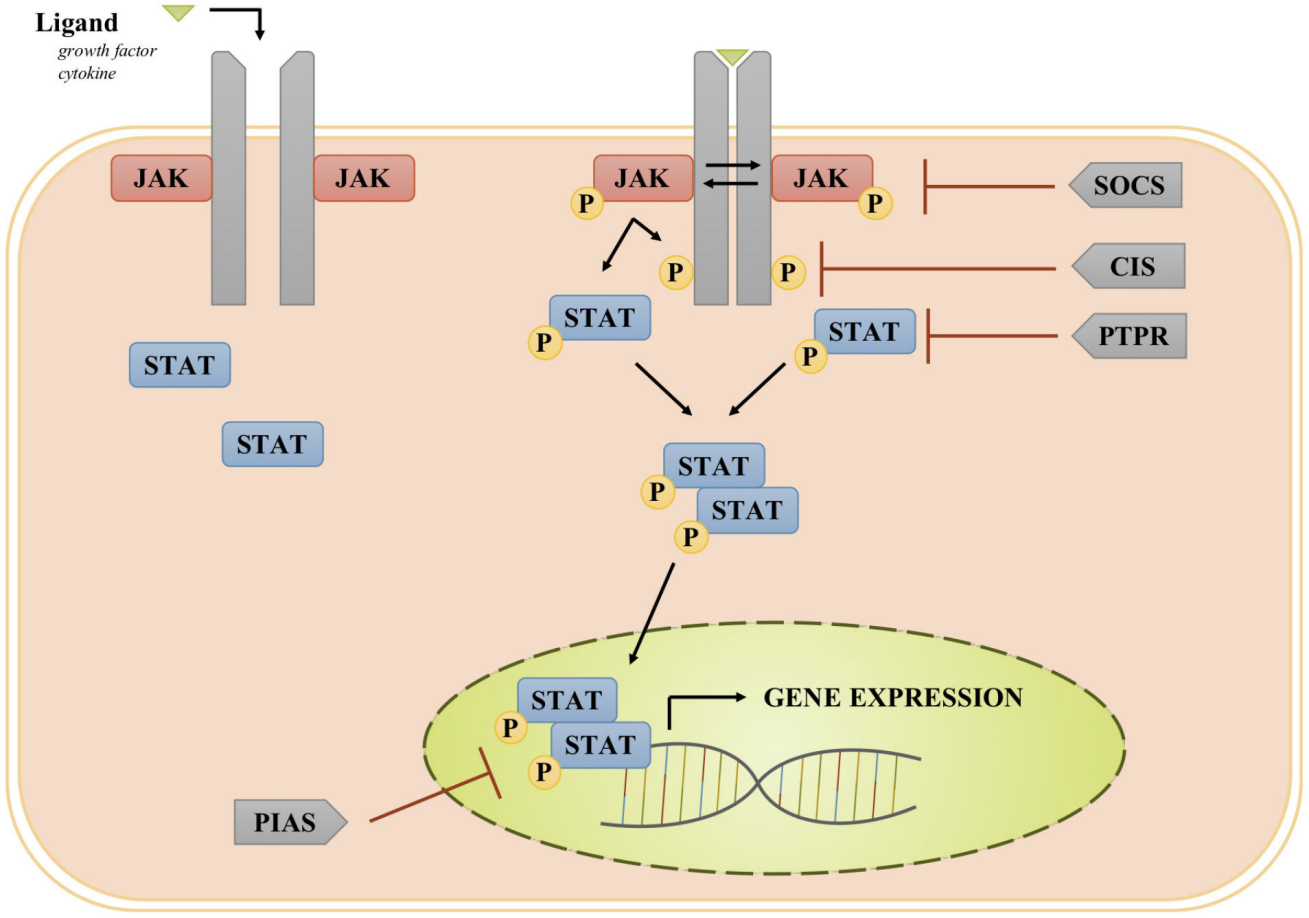
JAK/STAT pathway involving receptors lacking intrinsic tyrosine kinase activity: Upon ligand binding, transmembrane cytokine receptors multimerize, bringing receptor-associated JAKs into close physical proximity. Once activated via transphosphorylation, JAKs phosphorylate the cytoplasmic domain of the receptor to provide a docking site for STAT. The bound STATs are then phosphorylated and activated by JAKs. Activated STATs dimerize and translocate into the nucleus where act as transcription factors. Suppressors of cytokine signaling (SOCS) family of proteins inhibit JAK activation; cytokine-inducible SH2-containing protein (CIS) blocks the STAT docking site on the receptor; Protein inhibitor of activated STAT (PIAS) proteins inhibit STAT binding to promoter regions of target genes; and protein tyrosine phosphatase receptors (PTPRs) dephosphorylate STATs. JAKs: Janus kinases; STAT: signal transducer and activator of transcription

Ligands, particularly cytokines and growth factors, provide the initial stimulus for activating the JAK/STAT pathway^[23]^. Cytokines bind to their corresponding transmembrane receptor subunits, resulting in multimerization with other subunits and close physical interactions of receptor-associated JAKs^[24]^. The JAK family of tyrosine kinases consists of JAK1, JAK2, JAK3, and TYK2^[25]^. Once the receptor-associated JAKs are placed in close proximity, they become activated via trans-phosphorylation^[24]^. Activated JAKs phosphorylate tyrosine residues on the cytoplasmic region of the cytokine receptor to provide docking sites for the Src Homology 2 (SH2) domain of STAT proteins. The binding of a member of the STAT family of proteins (STAT1, STAT2, STAT3, STAT4, STAT5a, STAT5b, and STAT6) to the phosphorylated receptor intracellular domain results in JAK-mediated tyrosine phosphorylation and activation of the STAT protein [Figure 1]^[26]^. In the case of receptors with intrinsic tyrosine kinase activity (e.g., epidermal growth factor receptor, EGFR), ligand binding results in receptor autophosphorylation of tyrosine residues which serve as the docking sites for STATs, and the bound STATs are directly phosphorylated/activated by the receptor tyrosine kinase. Activated STATs dimerize and translocate into the nucleus where they serve as transcription factors, inducing the expression of genes that regulate cellular proliferation, survival, and invasion, as well as the host immune response^[14,24,27,28]^.

The JAK/STAT signaling pathway is modulated by several negative regulators^[29]^. Members of the suppressors of cytokine signaling (SOCS) family of proteins, such as SOCS1 and SOCS3, are inhibitory against JAKs, while cytokine-inducible SH2-containing protein (CIS) blocks STAT binding sites on receptor proteins^[29,30]^. Another family of inhibitory molecules, the protein inhibitor of activated STAT (PIAS) proteins, inhibit the binding of STATs to response elements in target genes^[29,31]^. Protein tyrosine phosphatase receptors (PTPRs), specifically PTPRT, PTPRD, and PTPRK, have been shown to dephosphorylate STAT3, resulting in STAT3 inactivation; a handful of non-receptor PTPs harbor a similar function^[32-37]^. Increased activity of JAKs and STATs coupled with decreased activity of negative regulators can lead to an upregulation of pro-proliferative, anti-apoptotic, and immunosuppressive proteins, potentially driving oncogenesis.

## DYSREGULATION OF THE JAK/STAT PATHWAY IN SOLID TUMORS

### Hyperactivation of STAT3

Hyperactivation of STATs, particularly STAT3, has been implicated in many cancers. Upstream JAK2 V617F mutations in myeloproliferative diseases and STAT3 mutations in T-cell large granular lymphocytic leukemia provide mechanisms for STAT3 hyperactivity in hematological malignancies^[15,38,39]^. JAK1 mutations have been identified in hepatocellular carcinoma (HCC) patient tumors; patient-derived xenografts with JAK1 S703I mutations had elevated levels of phosphorylated STAT3 and STAT5^[40,41]^. However, for most cases of solid tumors, activating mutations in this pathway have not been identified^[42]^.

In most solid tumors associated with hyperactivation of STAT3, disease development and progression has been attributed to either increased cytokine signaling or inhibition of negative regulators of the JAK/STAT pathway^[42,43]^. In head and neck cancers (HNC), aberrant activation of STAT3, often through elevated IL-6 levels in the tumor microenvironment, is associated with increased tumor cell proliferation, survival, and metastasis, as well as immunosuppression of tumor-infiltrating immune cells^[44-46]^. As in HNC, gastric cancer cell lines exhibit IL-6-mediated STAT3 activation, which leads to increased cell survival and epithelial to mesenchymal transition *in vitro*^[47,48]^. Gastric cancer tumors were also found to have increased levels of phosphorylated STAT3 compared to healthy tissue^[49]^. In non-small cell lung cancer (NSCLC), secretion of oncostatin-M (OSM), a member of the IL-6 cytokine family, by cancer-associated fibroblasts increases STAT3 activity through activation of JAK1 and is a possible mechanism of resistance to targeted therapy such as EGFR and MEK inhibitors^[50]^. STAT3 hyperactivity seen in pancreatic cancers has been associated with increased IL-22-induced STAT3 signaling and SOCS3 suppression, leading to increased invasion, migration, and angiogenesis^[51-53]^. PTPRT, another negative regulator of the JAK/STAT pathway, is silenced via promoter hypermethylation in many cases of HNC and provides a likely mechanism for STAT3 hyperactivation in this cancer^[54]^. Loss-of-function mutations in PTPRD have also been implicated^[55]^. Hyperactivation of STAT3 has been reported in many other solid tumor malignancies, including breast cancer, HCC, and ovarian cancer, among others^[56-58]^.

### Hyperactivation of other STATs

While less common, hyperactivation of other members of the STAT protein family has been shown in some solid tumors. STAT1 drives aromatase inhibitor resistance in breast cancer, and is highly expressed in estrogen receptor-positive, tamoxifen-resistant breast cancer cell lines, indicating it may be a promising target in this malignancy^[59]^. STAT2 is not only highly expressed in ovarian cancer compared to normal ovarian tissue, but is also associated with metastasis and poor overall survival^[60]^. STAT2 is also associated with poor overall survival in NSCLC^[61]^. Hyperactivity of STAT5 is associated with enhanced cell viability, tumor growth, and recurrence in prostate cancers^[62,63]^. In colorectal cancer cell lines, elevated levels of activated STAT6 are correlated with metastasis and decreased apoptosis^[64]^.

Collectively, there is ample evidence showing that increased JAK/STAT signaling is associated with increased cell proliferation, cell survival, immune evasion, recurrence, and drug resistance in solid tumors; this pathway therefore represents a promising target for therapeutic intervention.

## JAK INHIBITORS

**Figure 2.**
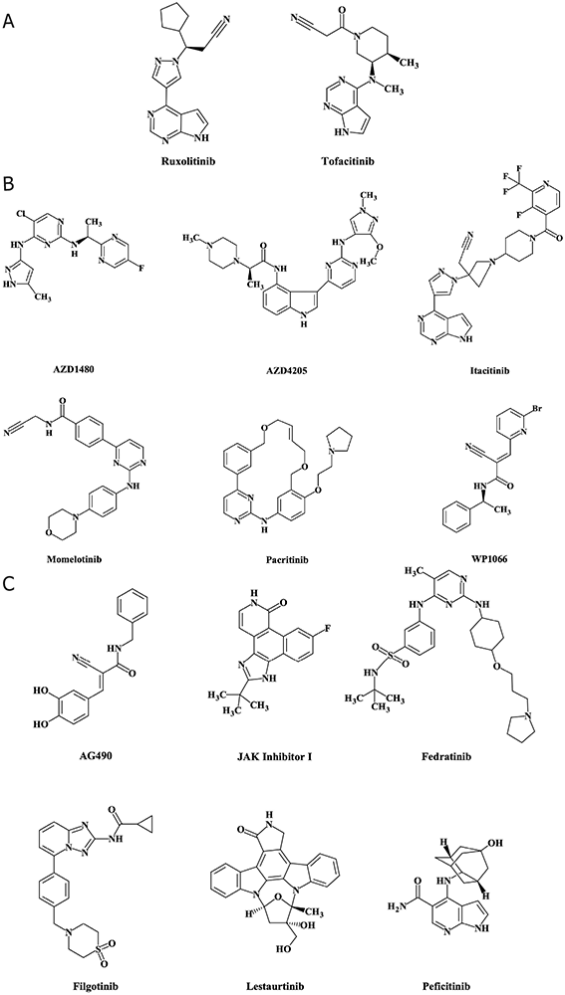
JAK inhibitors: chemical structures for JAK inhibitors described in this review were created using MarvinSketch software downloaded from ChemAxon (Budapest, Hungary)^[115,171-183]^. Food and Drug Administration (FDA)-approved JAK inhibitors that have been tested clinically in solid tumors (A); JAK inhibitors that are not FDA approved but have been tested clinically in solid tumors (B); JAK inhibitors that have only been tested in solid tumor preclinical models, and lestaurtinib, which has been tested clinically in solid tumors for its activity against other targets (C). Structures for INCB047986 and INCB052793 are not publicly available and therefore are not shown in this figure. *FDA approved for another indication; JAKs: Janus kinases

While hyperactivation of STATs, primarily STAT3, has been linked to the development and progression of solid tumors, STATs, similar to other transcription factors, have proven difficult to target directly. Therefore, upstream activators of STATs, such as JAKs, have been studied in preclinical and clinical settings as potential therapeutic targets. Several JAK inhibitors have been studied in solid tumors. Figure 2 depicts JAK inhibitors that are: (1) FDA approved and have been tested clinically in solid tumors [Figure 2A]; (2) not FDA approved, but have been tested clinically in solid tumors [Figure 2B]; and (3) have only been tested in solid tumor preclinical models. One multitarget agent (lestaurtinib) has been tested clinically for its activity against other targets [Figure 2C]. To date, there are 10 JAK inhibitors (two of which are FDA approved for other indications) that have been or are currently being investigated across 45 clinical trials in patients with solid tumors (excluding trials that have been withdrawn or in which JAK inhibitor was standard of care in studies investigating other agents) [Table 1]. Some compounds, a few of which are also FDA approved for other indications, have to date only been studied in solid tumor preclinical models.

### JAK inhibitors investigated in clinical trials

#### Ruxolitinib

The JAK1/2-selective inhibitor ruxolitinib is FDA approved for the treatment of polycythemia vera, myelofibrosis, and graft versus host disease, and it has been shown to decrease STAT3 activation in preclinical models of several solid tumors^[18,22,65]^. Ruxolitinib inhibited STAT3 activation and decreased cell growth in breast cancer^[66,67]^, NSCLC^[68]^, HNC^[69]^, esophageal cancer^[70]^, bladder cancer^[71]^, HCC^[72]^, cervical cancer^[73]^, and colorectal cancer^[74,75]^ cell lines. In pancreatic cancer cells, ruxolitinib treatment was also shown to decrease expression of pro-angiogenic genes and impede epithelial-to-mesenchymal transition^[76,77]^. In *in vivo* xenograft models of neuroblastoma^[78,79]^, HCC (in which there was a JAK1 S703I mutation)^[40]^, and KRAS-mutated lung adenocarcinoma^[80]^, among others, ruxolitinib treatment significantly inhibited tumor growth. Ruxolitinib treatment was associated with an increase in CD8+ T cells in pancreatic cancer xenograft models and a decrease in myeloid-derived suppressor cells in KRAS-mutated lung adenocarcinoma models, indicating an impact on immune activity^[52,80]^.

Ruxolitinib has also been shown to overcome drug resistance and increase sensitivity to several chemotherapeutic or targeted agents. In preclinical *in vitro* and *in vivo* models of cisplatin-resistant NSCLC, with increased JAK2 and STAT3 activation levels, the addition of ruxolitinib to cisplatin decreased STAT3 activation and cell growth, enhanced apoptosis, and inhibited tumor growth^[81]^. In myxoid liposarcoma cancer stem cells, which can be resistant to chemotherapy due to upregulated JAK/STAT signaling, ruxolitinib treatment inhibited phosphorylation of STAT3 and cell viability, overcoming chemotherapy resistance^[82]^. Ruxolitinib in combination with antibodies against cytokines such as IL-6 (tocilizumab) improved survival in mice bearing ovarian cancer tumors. Ruxolitinib in combination with paclitaxel reduced cell proliferation and colony formation in ovarian cancer cell lines and inhibited tumor growth in *in vivo* models^[83,84]^. Ruxolitinib has been shown to improve sensitivity to oncolytic viral therapy in HNC^[85]^, pancreatic cancer^[86]^, glioblastoma multiforme (GBM)^[87]^, and NSCLC^[88]^. Collectively, the safety profile of ruxolitinib in conjunction with promising preclinical findings in a variety of tumor models make ruxolitinib an attractive therapeutic agent against solid tumors.

Several clinical trials have studied the impact of ruxolitinib in patients with solid tumors. In a Phase II study of ruxolitinib and capecitabine in patients with pancreatic cancer who failed to respond to gemcitabine, known as the RECAP trial, there was improved survival among a subgroup of patients with inflammation, defined by a C-reactive protein (CRP) greater than the population median of 13 mg/L (NCT01423604)^[89]^. Given these initial promising results, ruxolitinib was administered to patients with pancreatic cancer and an elevated CRP in two Phase III trials, JANUS 1 (NCT02117479) and JANUS 2 (NCT02119663). In both trials, patients were randomized to be treated with either ruxolitinib and capecitabine or placebo and capecitabine. However, these studies were terminated as there was no increase in overall or progression-free survival observed in the group receiving ruxolitinib compared with placebo^[90]^. The combination of ruxolitinib and capecitabine in breast cancer patients with elevated CRP was also investigated in a Phase II clinical trial (NCT02120417). While patients receiving ruxolitinib and capecitabine had a more favorable health-related quality of life outcome, this study was terminated because there was no improvement in overall survival compared to the group receiving placebo and capecitabine^[91]^. A Phase II trial of ruxolitinib in triple-negative breast cancer confirmed inhibition of STAT3 activation in patient tumor samples; however, no clinical response was observed, as evaluated by the RECIST criteria, and the study was terminated (NCT01562873)^[92,93]^. The most recently completed clinical trial (results not reported or published) included a Phase II study testing ruxolitinib in combination with exemestane in patients with estrogen receptor-positive breast cancer (NCT01594216). The addition of ruxolitinib to regorafenib in a Phase II trial in patients with colorectal cancer did not show a difference in overall survival or progression-free survival as compared to placebo and regorafenib; therefore, this study was terminated early (NCT02119676)^[94]^. Ruxolitinib was also tested in patients with lung cancers. A Phase II trial of ruxolitinib (or placebo), pemetrexed, and cisplatin in patients with stage IIIb/IV or recurrent NSCLC demonstrated that this combination was well-tolerated; the study was terminated without achieving an efficacy endpoint (NCT02119650)^[95]^. Partial responses were seen in 31% of patients who received ruxolitinib and in 35% of patients who received placebo. A Phase Ib study of ruxolitinib combined with afatinib, an inhibitor of mutant EGFR, in patients with NSCLC showed that this regimen was both well-tolerated and displayed activity against this malignancy, as 23.3% displayed a partial response and 80% had stable disease (NCT02145637)^[96]^. In a Phase I/II study, ruxolitinib combined with the EGFR inhibitor erlotinib in lung adenocarcinoma was shown to be well-tolerated but ineffective (NCT02155465)^[97]^. A Phase Ib study of ruxolitinib with gemcitabine or nab-paclitaxel in solid tumors showed that this combination was well-tolerated (NCT01822756). However, efficacy could not be evaluated due to early termination of the trial after results from JANUS 1 showed no benefit of ruxolitinib and capecitabine compared to placebo and capecitabine^[98]^. In a Phase II trial of ruxolitinib in metastatic prostate cancer, there was no significant clinical response and the trial was terminated (NCT00638378). There is currently a rollover study that is providing continued access to ruxolitinib for patients with pancreatic, colorectal, lung, and breast cancers enrolled in previous trials (NCT02955940).

Several ongoing early-stage clinical trials are investigating ruxolitinib as monotherapy. There are two current window-of-opportunity trials: one testing neoadjuvant ruxolitinib in HNC (NCT03153982) and one examining ruxolitinib in premalignant breast disease (NCT02928978). Some trials are also investigating ruxolitinib in combination other agents. Among these are a Phase I study testing ruxolitinib in combination with temozolomide in patients with high-grade gliomas (NCT03514069), a Phase Ib study of ruxolitinib and trametinib (MEK inhibitor) in colon and pancreatic cancers with RAS mutations (NCT04303403), a Phase I study testing ruxolitinib with pembrolizumab (PD-L1 inhibitor) in triple-negative breast cancer (NCT03012230), two Phase II studies investigating ruxolitinib with chemotherapy in inflammatory breast cancer (NCT02876302, NCT02041429), a Phase I/II trial evaluating ruxolitinib with trastuzumab (HER2 inhibitor) in HER2+ breast cancer (NCT02066532), and a Phase I/II study of ruxolitinib with paclitaxel and carboplatin in ovarian, fallopian tube, and peritoneal cancers (NCT02713386). Ruxolitinib is one of 75 approved agents being tested in a trial that uses the Co-eXpression ExtrapolatioN (COXEN) model to identify biomarkers and to predict which drugs would provide the most benefit to patients with urothelial cancer (NCT02788201).

#### Tofacitinib

Tofacitinib is a JAK1/3 inhibitor that is FDA approved for treatment of rheumatoid arthritis and ulcerative colitis^[99-102]^. Tofacitinib treatment of breast cancer cells prevented activation and nuclear localization of STAT3^[103]^. In prostate cancer preclinical models, tofacitinib decreased STAT5 activation and epithelial-to-mesenchymal transition^[104]^. This JAK inhibitor is currently being tested in patients with solid tumors (mainly pancreatic adenocarcinoma and cholangiocarcinoma) in a Phase I trial (NCT04034238).

#### AZD1480

AZD1480 is a selective ATP-competitive JAK1/2 inhibitor that showed promising activity against many solid tumor preclinical models. AZD1480 treatment of cell lines and murine models, including but not limited to GBM^[105]^, breast cancer^[106,107]^, HNC^[108]^, and ovarian cancer^[109]^, inhibited STAT3 activation, cell viability, and tumor growth. Despite these encouraging preclinical findings, neurotoxicity was observed in a Phase I clinical trial of AZD1480 in solid tumors and halted the development of this agent, leading to the termination of this trial (NCT01112397) and a parallel Phase I study in patients with HCC, NSCLC, and gastric cancer (NCT01219543)^[110]^.

#### AZD4205

AZD4205 is a selective JAK1 inhibitor^[111]^. In a preclinical NSCLC *in vivo* model, AZD4205 treatment inhibited tumor growth and STAT3 activation; these findings were more significant when AZD4205 was administered in combination with the EGFR inhibitor osimertinib. A Phase I/II clinical trial investigating AZD4205 combined with osimertinib was initiated in patients with NSCLC (NCT03450330).

#### INCB047986 and INCB052793

INCB047986 and INCB052793 are selective inhibitors of JAK1. INCB047986 was studied in a Phase I clinical trial in breast and pancreatic cancers, among other solid tumors, but the trial was terminated early (NCT01929941). INCB052793 has been studied in multiple myeloma (MM) preclinical models, but there are no reports using this agent in solid tumors. In combination with other anti-MM agents, INCB052793 decreased cell viability and inhibited tumor growth^[112]^. A Phase I/II trial was initiated investigating this agent in solid tumors but was terminated due to lack of efficacy (NCT02265510).

#### Itacitinib

Preclinical studies of the JAK1 inhibitor itacitinib have mostly been conducted in preclinical models of hematological malignancies. In conjunction with INCB054329, an inhibitor of bromodomain and extra-terminal motif proteins, itacitinib inhibited STAT3 activation and tumor growth in MM cell lines and murine models^[113]^. Given this effect of JAK1 inhibition on STAT3 activity, clinical studies with this agent were initiated in solid tumors. In a Phase Ib/II study of itacitinib in combination with nab-paclitaxel and gemcitabine in solid tumors (84% of which had pancreatic cancer), 24% of patients responded (all partial response) (NCT01858883). The therapeutic combination was well-tolerated after dose reduction of itacitinib; however, this study was terminated due to another Phase III clinical trial reporting no impact of the JAK1/2 inhibitor ruxolitinib on overall survival in pancreatic cancer^[114]^. Other ongoing clinical trials are studying itacitinib in patients with HCC (NCT04358185), NSCLC (NCT03425006 and NCT02917993), and a variety of advanced solid tumors (NCT02646748). A Phase II study in soft tissue sarcoma is currently suspended (NCT03670069); a Phase II study in NSCLC (NCT02257619) and a Phase Ib study in other solid tumors (NCT02559492) were terminated, with no published findings.

#### Momelotinib

Momelotinib is a JAK1/2 inhibitor that also has activity against TANK-binding kinase 1 (TBK1)^[115,116]^. Several preclinical studies in solid tumor models have investigated the impact of momelotinib on the JAK/STAT pathway. Momelotinib has been shown to increase sensitivity of ovarian cancer to chemotherapy in *in vitro* and *in vivo* preclinical models^[117,118]^. In combination with paclitaxel, momelotinib inhibited tumor growth, suppressed STAT3 activation, reduced expression of the stem cell marker OCT4, significantly increased the time to recurrence, and decreased tumor burden^[117,118]^. Similarly, in GBM preclinical models, momelotinib in combination with temozolomide inhibited STAT3 activation, decreased cell growth, increased apoptosis, and inhibited tumor growth compared to temozolomide monotherapy^[119]^. In colorectal cancer cells, momelotinib inhibited STAT5 activation, decreased cell growth, and increased cell death^[120]^. These promising preclinical results across several types of solid tumors support further investigation of momelotinib as a therapeutic agent.

Clinical use of momelotinib has been studied extensively in myeloproliferative diseases: in myelofibrosis, treatment with this agent was associated with a reduction in splenic volume that was non-inferior to ruxolitinib^[121]^. Its impact in solid tumors is under active clinical investigation. In a Phase I dose-escalation study in patients with untreated metastatic pancreatic cancer, momelotinib in combination with gemcitabine and nab-paclitaxel was well-tolerated; however, limited efficacy and no apparent association between efficacy and increasing dose led to the termination of this trial prior to the initiation of planned Phase III studies (NCT02101021)^[122]^. A Phase Ib study of momelotinib combined with trametinib in KRAS-mutated NSCLC showed no improvement in response compared with historic data with trametinib monotherapy (NCT02258607)^[123]^. In a Phase Ib study of momelotinib in combination with erlotinib in EGFR-mutated, metastatic NSCLC, patients experienced neutropenia as an adverse effect of this drug combination, and the trial was halted (NCT02206763)^[124]^. Another Phase Ib clinical trial of momelotinib with chemotherapeutic agents, capecitabine and oxaliplatin, in pancreatic adenocarcinoma was terminated (NCT02244489).

#### Pacritinib

Pacritinib is a selective JAK2 inhibitor currently being studied in a Phase III clinical trial for treatment of myelofibrosis (NCT02055781)^[125,126]^. In GBM cell lines, pacritinib, alone or in combination with afatinib, inhibited STAT3 activation, cell viability, and spheroid formation^[127-129]^. Pacritinib plus afatinib was also shown to decrease tumor burden in mice with GBM tumors^[129]^. Similar to momelotinib, pacritinib reduced resistance to temozolomide in GBM *in vivo* models^[127,128]^. Pacritinib has been shown to inhibit liver fibrosis and thus may be effective in preventing HCC^[130]^. A Phase II trial of pacritinib in refractory colorectal cancers is ongoing (NCT02277093)^[131]^. Pacritinib was also studied in combination with erlotinib in a Phase I/II trial in NSCLC, which was terminated (NCT02342353).

#### WP1066

WP1066 inhibits JAK2 phosphorylation and causes JAK2 degradation; it is an analog of the JAK2 inhibitor AG490, an agent which was widely tested in preclinical modes of solid tumors^[132,133]^. Preclinical studies have shown that WP1066 exhibits anti-cancer activity including inhibition of cell proliferation and survival, and/or inhibition of tumor growth in solid tumors including, but not limited to, bladder cancer, renal cell carcinoma (in which it was shown to inhibit angiogenesis), HNC, GBM, and NSCLC^[134-139]^. This agent also inhibited migration and invasion in bladder cancer, hepatocellular carcinoma, and GBM cell lines^[134,139,140]^. WP1066 treatment overcame STAT3-mediated cisplatin resistance in oral squamous cell carcinoma and ovarian cancer cell lines, and doxorubicin resistance in breast cancer cell lines^[141-143]^. Two current Phase I trials investigating the safety and efficacy of WP1066 are being conducted in pediatric medulloblastomas (NCT04334863) and adult malignant gliomas or brain metastases (NCT01904123).

### JAK inhibitors with preclinical evidence supporting activity against solid tumors

While several JAK inhibitors have not yet been tested in patients with solid tumors, they have shown promising anti-cancer effects in preclinical models. Agents such as AG490, the compound from which WP1066 was derived, and JAK inhibitor I have been widely tested in preclinical *in vitro* and *in vivo* models. AG490 inhibited STAT3 activation and exhibited anti-cancer effects such as inhibition of cell growth and induction of apoptosis via targeting of JAK2 in preclinical models of breast cancer^[4]^, gastric cancer^[48]^, pancreatic cancer^[144]^, and gallbladder cancer^[145]^, among others. JAK inhibitor I is a JAK1/2/3 inhibitor that decreased cell proliferation in breast cancer cells^[146]^, increased apoptosis in esophageal squamous cell carcinoma cancer stem cells^[147]^, inhibited STAT3 phosphorylation in HCC cells^[148]^, and, in combination with cisplatin, decreased PD-L1 expression in prostate cancer cells^[149]^. In addition to these agents, there are a handful of inhibitors that have either already been FDA approved or are being tested currently in clinical trials for other indications and have also shown promising findings in solid tumor preclinical models. The following inhibitors, therefore, are potential candidates for clinical testing and use in patients with solid tumors.

#### Fedratinib

Fedratinib is an orally bioavailable, small molecule, JAK2 inhibitor that is FDA approved for the treatment of myelofibrosis^[150-153]^. NSCLC cells have been shown to be sensitive to fedratinib; sensitivity was shown to be correlated with elevated JAK2 expression^[154]^. Two studies showed that fedratinib in combination with erlotinib (EGFR tyrosine kinase inhibitor) decreased STAT3 activation and increased apoptosis in erlotinib-resistant NSCLC cells and inhibited tumor growth in *in vivo* murine models^[155,156]^. This agent has also demonstrated cell-killing activity against ovarian and cervical cancer cells^[157]^. Fedratinib inhibited mammosphere formation and in combination with carboplatin, inhibited breast cancer tumor growth in mice^[158]^. In human papilloma virus (HPV)-positive cervical cancer cells, fedratinib treatment inhibited JAK2 and STAT3/5 activation, increased apoptosis, and reduced cyclin D1 expression, cell proliferation, and colony formation^[73]^. In HNC cells, treatment with fedratinib increased susceptibility to natural killer cell killing^[159]^.

#### Filgotinib

Filgotinib is a selective JAK1 inhibitor currently being investigated in clinical trials for treatment of rheumatoid arthritis and inflammatory bowel disease; to date, this drug demonstrates a significant anti-inflammatory effect, as it reduces levels of cytokines such as IL-6^[160-162]^. Findings from preclinical studies in solid tumors have been reported. The OSM-JAK-STAT pathway has been implicated in progression of several cancers, including NSCLC; treatment of NSCLC cells with filgotinib resulted in inhibition of STAT3 activation and reduced OSM receptor expression^[50]^. Furthermore, treatment with filgotinib inhibited resistance to targeted therapy such as MEK, EGFR tyrosine kinase, and ALK inhibitors. In NCI-H889 lung cancer cells, derived from a metastatic site, filgotinib inhibited STAT3 activation^[163]^. *In vivo*, filgotinib treatment reduced metastatic seeding of NCI-H889-derived tumors. In a breast cancer cell line, filgotinib inhibited STAT3 phosphorylation; in combination with a histone deacetylase inhibitor, there was increased apoptosis in breast cancer cells as well as tumor growth inhibition in mice harboring breast cancer tumors^[164]^.

### Lestaurtinib

Lestaurtinib is a multitarget inhibitor that has activity against JAK2, in addition to fms-like tyrosine kinase tyrosine 3 (FLT3) and tropomyosin related kinase B (TrkB)^[165]^. Its impact on the JAK/STAT pathway has been studied clinically in myeloproliferative disorders, but trials in solid tumors such as neuroblastoma focus on its activity against other targets such as TrkB. One preclinical study showed that lestaurtinib treatment of anaplastic thyroid cancer cell lines inhibited STAT5 phosphorylation/activation, cell proliferation, cell survival, and cell migration, in addition to tumor growth in *in vivo* models^[166]^.

### Peficitinib

Peficitinib is a JAK1/2/3 and TYK2 inhibitor, approved in Japan in 2019 for rheumatoid arthritis after Phase III clinical trials demonstrated a reduction in symptoms and minimal toxicity compared to placebo in clinical trials^[167,168]^. Only one study to date has reported its potential use in solid tumors^[169]^. In ovarian cancer stem cells engineered to overexpress OCT4, peficitinib induced apoptosis and inhibited proliferation in conjunction with JAK1 inhibition.

## CONCLUSION

Aberrant JAK/STAT signaling is associated with solid tumor development and progression. However, unlike hematopoietic malignancies which harbor activating JAK mutations that lead to increased JAK/STAT signaling, the majority of solid tumors that demonstrate increased JAK/STAT signaling lack somatic JAK mutations. Studies in preclinical cancer models of solid tumors collectively show that small molecule JAK inhibitors inhibit activation of STATs, particularly STAT3, in conjunction with inhibition of proliferation and tumor growth. The majority of JAK inhibitors tested in clinical trials, with the exception of AZD1480, were found to be safe and well-tolerated. Among these, ruxolitinib is the only inhibitor to date to demonstrate responses in early stage trials. While Phase II trial results in pancreatic cancer suggested an association between elevated CRP and response to ruxolitinib plus capecitabine, these findings were not seen in the Phase III trials^[89,90]^. In patients with elevated CRP, ruxolitinib combined with capecitabine was associated with improved health-related quality of life in breast cancer^[91]^. Additionally, treatment of patients with NSCLC with ruxolitinib plus afatinib resulted in partial responses and stable disease^[96]^. However, most trials testing ruxolitinib exhibited disappointing results, and several were terminated early; this could possibly be explained by JAK inhibition impeding immune cell function, which may counteract some of the drug’s other anti-cancer effects^[170]^. It is clear that only a subset of solid tumors is likely to be sensitive to JAK inhibition. Candidate predictive biomarkers to date include elevated CRP in pancreatic and breast cancers, PTPRT/D mutations in HNC, and a JAK1 S703I mutation in HCC, and assessments of biologically plausible biomarkers that predict clinical responses are needed^[40,54,55,89,91]^. The JAK inhibitors fedratinib, filgotinib, and peficitinib have been shown to abrogate JAK/STAT signaling and induce anti-tumor effects in solid tumor cell lines, but, to date, there are no clinical trials investigating these agents in solid tumors; lestaurtinib has been tested clinically in solid tumors for its activity against other targets not directly involved in the JAK/STAT pathway. Ruxolitinib, tofacitinib, fedratinib, and peficitinib are JAK inhibitors already approved for other indications, making them especially attractive options as they are known to be well-tolerated. Further investigation of JAK inhibitors in clinical trials is warranted to determine the therapeutic potential in solid tumors.

## Figures and Tables

**Table 1.** JAK inhibitors tested clinically in solid tumors

Inhibitor	Direct Target(s)	NCT#	Type(s) of solid tumor	Status	Outcome
Ruxolitinib*	JAK1/2	NCT01423604	PDAC	Completed	Improved overall survival in subgroup of patients with inflammation^[89]^
		NCT02117479	PDAC	Terminated	No overall survival benefit^[90]^
		NCT02119663	PDAC	Terminated	No overall survival benefit^[90]^
		NCT02120417	BC	Terminated	Favorable HRQoL, no overall survival benefit^[91]^
		NCT01562873	BC	Terminated	No tumor response^[92]^
		NCT01594216	BC	Completed	None published
		NCT02119676	CRC	Terminated	No overall survival benefit^[94]^
		NCT02119650	NSCLC	Terminated	Unable to interpret efficacy^[96]^
		NCT02145637	NSCLC	Completed	23.3% PR, 70.0% SD^[96]^
		NCT02155465	Lung adenocarcinoma	Completed	Lack of efficacy^[97]^
		NCT01822756	Advanced solid tumors	Terminated	Unable to interpret efficacy^[98]^
		NCT00638378	PC	Terminated	Lack of clinical response
		NCT02955940	PDAC, CRC, BC, NSCLC	Active	
		NCT03153982	HNC	Active	
		NCT02928978	Premalignant breast disease	Active	
		NCT03514069	High-grade gliomas	Active	
		NCT04303403	CRC, PDAC	Active	
		NCT03012230	BC	Active	
		NCT02876302	IBC	Active	
		NCT02041429	IBC	Active	
		NCT02066532	BC	Active	
		NCT02713386	OC, fallopian tube cancer, peritoneal cancer	Active	
		NCT02788201	UC	Completed	None published
Tofacitinib*	JAK1	NCT04034238	Epithelioid mesothelioma, cholangiocarcinoma, PDAC	Active	
AZD1480	JAK1/2	NCT01112397	Advanced solid tumors, not specified	Terminated	pSTAT3 inhibition in granulocytes, neurotoxicity in patients^[110]^
		NCT01219543	HCC, NSCLC, GC	Terminated	None published
AZD4205	JAK1	NCT03450330	NSCLC	Completed	None published
INCB047986	JAK1	NCT01929941	PDAC, BC, non-specified advanced solid tumors	Terminated	None published
INCB052793	JAK1	NCT02265510	Non-specified advanced solid tumors	Terminated	Lack of efficacy
Itacitinib	JAK1	NCT01858883	Variety (84% PDAC)	Completed	Lack of efficacy in JANUS 1 and JANUS 2 trials^[114]^
		NCT04358185	HCC	Active	
		NCT03425006	NSCLC	Active	
		NCT02917993	NSCLC	Active	
		NCT02646748	CRC, endometrial cancer, HNC, lung cancer, BC, PDAC, RCC, UC	Active	
		NCT03670069	Soft tissue sarcoma	Suspended	
		NCT02257619	NSCLC	Terminated	None published
		NCT02559492	Non-specified advanced solid tumors	Terminated	None published
Momelotinib	JAK1/2	NCT02101021	PDAC	Terminated	No overall survival benefit^[122]^
	TBK1	NCT02258607	NSCLC	Terminated	No overall survival benefit^[123]^
		NCT02206763	NSCLC	Terminated	Neutropenia^[124]^
		NCT02244489	PDAC	Terminated	None published
Pacritinib	JAK2	NCT02277093	CRC	Terminated	Lack of clinical response
		NCT02342353	NSCLC	Terminated	None published
WP1066	JAK2	NCT04334863	Medulloblastoma, brain metastases	Active	
		NCT01904123	Glioma, brain metastases	Active	

This table summarizes active, completed, or terminated clinical trials registered in ClinicalTrials.gov of JAK inhibitors in solid tumors. Outcomes of the studies were reported in published articles describing the trials or in the study descriptions at ClinicalTrials.gov.

* Agents FDA-approved for other indication. JAK: Janus kinase; PDAC: pancreatic ductal adenocarcinoma; BC: breast cancer; HRQoL: health-related quality of life; CRC: colorectal cancer; NSCLC: non- small cell lung cancer; PR: partial response; SD: stable disease; PC: prostate cancer; HNC: head and neck cancer; IBC: inflammatory breast cancer; OC: ovarian cancer; UC: urothelial cancer; pSTAT3: phosphorylated signal transducer and activator of transcription 3; HCC: hepatocellular carcinoma; GC: gastric cancer; RCC: renal cell carcinoma; TBK1: TANK-binding kinase 1
